# Supplementary material for: Effects on the Physical Functioning of Two Exercise Interventions in Patients with Multiple Myeloma: A Pilot Feasibility Study
Source: Cancers (Basel). 2024 May 4;16(9):1774. doi: 10.3390/cancers16091774 (PMC11083081; doi:10.3390/cancers16091774)
Supplement: Supplementary file 1 [file cancers-16-01774-s001.zip › cancers-2914749-supplementary.pdf]

**Supplemental Table S1**. Adverse Event and Serious Adverse Event Reporting Categories
**MedDRA Primary SOC**
Blood and lymphatic system disorders
Cardiac disorders
Congenital, familial and genetic disorders
Ear and labyrinth disorders
Endocrine disorders
Eye disorders
Gastrointestinal disorders
General disorders and administration site conditions
Hepatobiliary disorders
Immune system disorders
Infections and infestations
Injury, poisoning and procedural complications
Investigations
Metabolism and nutrition disorders
Musculoskeletal and connective tissue disorders
Neoplasms benign, malignant and unspecified (incl cysts and polyps)
Nervous system disorders
Pregnancy, puerperium and perinatal conditions
Psychiatric disorders
Renal and urinary disorders
Reproductive system and breast disorders
Respiratory, thoracic and mediastinal disorders
Skin and subcutaneous tissue disorders
Social circumstances
Surgical and medical procedures
Vascular disorders
MedDRA=Medical Dictionary for Regulatory Activities; SOC=System Organ Class, from the Common Terminology Criteria for Adverse Events (CTCAE) Version 5.0

Supplemental Table S2. Adverse Event and Serious Adverse Event Grading and Relationship Structure

**CTCAE Grade**	**Description**
Grade 1	Mild; asymptomatic or mild symptoms; clinical or diagnostic observations only; intervention not indicated.
Grade 2	Moderate; minimal, local or noninvasive intervention indicated; limiting age-appropriate instrumental ADL.
Grade 3	Severe or medically significant but not immediately life-threatening; hospitalization or prolongation of hospitalization indicated; disabling; limiting self-care ADL.
Grade 4	Life-threatening consequences; urgent intervention indicated.
Grade 5	Death related to AE.
**Relationship to AE**	**Relationship Group**
Unrelated	Unrelated
Unlikely	
Possible	Related
Probable	
Definite	

CTCAE=Common Terminology Criteria for Adverse Events, Version 5.0; ADL=activities of daily living; AE=adverse event
